# Care Pathways and Patient Experiences Among Patients With Post COVID-19 Condition: Study Protocol for a Mixed-Methods Study in Germany

**DOI:** 10.2196/91976

**Published:** 2026-04-22

**Authors:** Daniela Gesell, Paula Lienesch, Yanyan Shi, Ralf Strobl, Martin Tauscher, Roman Gerlach, Eva Grill, Daniela Koller

**Affiliations:** 1 Department of Orthopaedics and Trauma Surgery, Musculoskeletal University Center Munich (MUM), University Hospital, LMU Munich, Marchioninistr. 15, Munich, 81377, Germany; 2 Institute of Medical Information Processing, Biometry and Epidemiology, Faculty of Medicine, LMU Munich, Marchioninistr 15Munich, Bavaria, 81377, Germany, 49 894400, 77376; 3 Institute for Medical Biometry, Informatics and Epidemiology, University Hospital Bonn, University of Bonn, Bonn, Germany; 4 German Center for Vertigo and Balance Disorders, LMU University Hospital, Munich, Germany; 5 Bavarian Association of Statutory Health Insurance Physicians, Munich, Germany

**Keywords:** PCC, long COVID, ambulatory care, healthcare pathways, patient experiences, mixed-methods, post COVID condition

## Abstract

**Background:**

The COVID-19 pandemic has a lasting impact on health care utilization, as both the acute infection and post COVID condition (PCC) can lead to increased demand for medical services due to ongoing symptoms.

**Objective:**

The aim of this study is to systematically examine health care utilization among individuals after acute SARS-CoV-2 infection in Bavaria, Germany, with a particular focus on PCC. The study combines claims data analysis with qualitative interviews to improve the understanding of objective care pathways and patients’ subjective experiences within the health care system.

**Methods:**

The research project ‘SOLongCOVID’ employs a mixed-methods design consisting of two subprojects: (1) a retrospective cohort study using claims data from the Bavarian Association of Statutory Health Insurance Physicians (KVB) to analyze care pathways through state sequence analysis, (2) a qualitative study based on semistructured interviews and focus groups with patients with PCC concerning their subjective care experiences. A synthesis process involving a focus group discussion will combine the information from the two subprojects, providing a comprehensive understanding of the care processes of patients with PCC.

**Results:**

The study was funded by the German Federal Joint Committee Innovation Fund in October 2024. Statutory health insurance claims data cover the period from 2019 to 2022, and qualitative interview data collection is planned from May 2025 to August 2026. As of manuscript submission, study preparation and ethics approvals have been completed, and 14 participants have been recruited for the qualitative interviews. Study findings are anticipated to be published from July 2026 to August 2027.

**Conclusions:**

The results are expected to enhance the understanding of existing barriers and challenges and to support evidence-based recommendations for improving care pathways for patients with specific care needs.

## Introduction

According to the World Health Organization, more than 770 million people worldwide have been infected with Severe Acute Respiratory Syndrome Coronavirus 2 (SARS-CoV-2), with approximately 6.9 million deaths [1]. While symptoms often subside within 14 days, many patients continue to have symptoms after the acute illness [2,3]. The generally used term ‘long COVID,’ medically described as *post-COVID-19 condition* (PCC) is defined as the presence of symptoms that persist or newly develop three months after a SARS-CoV-2 infection and last for at least two months without an alternative explanation [4]. The symptoms experienced by patients with PCC are highly diverse and reflect the multifaceted nature of the condition. These symptoms can affect multiple systems of the body, further highlighting the complexity of the condition [3,5,6]. However, there are certain symptoms that occur more frequently. These include fatigue, myalgia, neurological symptoms such as cognitive impairment, loss of smell and taste, as well as headaches, mental health issues, including depression and anxiety, and cardiopulmonary symptoms like dyspnea, chest pain, and palpitations [5,7-11].

The broad range and duration of these symptoms, combined with the diverse nature and unpredictability of PCC, often lead to increased and prolonged health care utilization in both ambulatory and hospital settings, as demonstrated in several studies [12-15]. The increase in utilization has a major impact on the health care system, which is faced with rising costs and increasing demand for specialized treatment [14,16,17]. A study from the United States showed that SARS-CoV-2 infection was associated with an overall 4% increase in health care utilization within six months of infection, primarily driven by virtual contacts and emergency department visits [18]. Specifically for the ambulatory setting, recent studies on PCC health care utilization have mainly focused on treatment by specialists or consultation with primary care physicians. However, many of these studies are limited by follow-up periods of less than six months [5,8,10,11]. At the same time, international research is increasingly addressing potential barriers to care and patient experiences in accessing PCC-related health care services. These studies indicate that access to care is often limited by long waiting times, a shortage of specialists, and a lack of specialized treatment services. They also underscore the urgent need for patient-centered approaches to improve care for those affected [19-23].

Evidence on long-term health care utilization, particularly with respect to different types of physician services, remains limited. Gaining a more comprehensive understanding of health care utilization and the specific needs of patients following a SARS-CoV-2 infection is essential for policymaking and clinical management of affected patients.

To improve the understanding of both objective care pathways and subjective patient experiences, claims data derived from the routine records of the Bavarian Association of Statutory Health Insurance Physicians (KVB) will be analyzed, and qualitative interviews and a focus group will be conducted to examine the individual care experiences of people affected by PCC. The aim of the study is to systematically examine health care utilization among patients after an acute SARS-CoV-2 infection, in Bavaria, Germany, with a particular focus on PCC. The study combines claims data analysis with qualitative interviews to improve the understanding of objective care pathways and patients’ subjective experiences within the health care system.

## Methods

To ensure a high scientific standard and transparency, the reporting of this study protocol and the study results will adhere to the following guidelines: the *Strengthening the Reporting of Observational Studies in Epidemiology* (STROBE) guideline and the *Consolidated criteria for reporting qualitative research* (COREQ) Checklist [24-26]. The study is registered in the German Clinical Trials Register (registration number DRKS00038446).

### Ethical Considerations

The claims data analysis and the qualitative study were approved by the Local Research Ethics Committee of Ludwig-Maximilians-University Munich (reference number 25‐0173-KB and 25‐0276). Written informed consent is obtained from each participant after they had been informed about the study’s aims, procedures, voluntary nature, and data protection measures. Participant privacy and confidentiality are strictly maintained throughout the study. Personal identifiers are removed or not collected where not essential, and all data are handled and stored securely in accordance with institutional ethics and data protection guidelines. No identifiable personal information is reported in this manuscript. Copies of the signed consent forms are securely stored in accordance with institutional ethics guidelines. No compensation is provided to participants.

### Study Design

**Figure 1.**
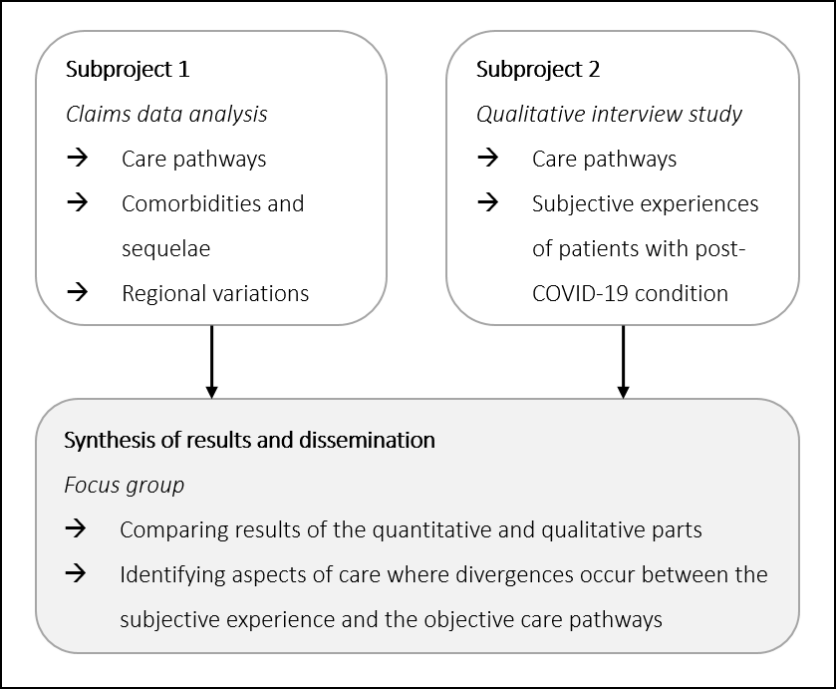
Mixed-methods study approach.

The study follows a convergent mixed-methods design aiming to describe and analyze the trajectories of patients following an acute SARS-CoV-2 infection in the ambulatory setting (see Figure 1). Quantitative analyses of statutory health insurance claims data and qualitative interview analyses will be conducted in parallel and independently. Data and results from two subprojects will be used to facilitate the synthesis.

First, in a retrospective cohort study with claims data (Subproject 1) objective care pathways will be reconstructed based on routine data from the KVB. To do so, three cohorts will be defined, a cohort of patients with SARS-CoV-2 infection and a cohort of patients with other respiratory tract infection during the pandemic phase 2020 till 2022. To measure the impact that the pandemic had on the overall health care utilization, we will also form a historic control group with respiratory tract infections in the year 2019.

Second, a qualitative study (Subproject 2) will be conducted, including semistructured interviews with people affected by PCC concerning their individual subjective experiences and personal motivations for (non-) utilization of care.

The integration of the results will take place at the interpretation stage through systematic triangulation. Quantitatively identified care pathways will be compared with themes derived from patient interviews to identify areas of convergence, complementarity, and divergence. Joint displays will be developed that compare quantitative care trajectories with qualitative interview data, enabling structured comparisons across data sources. This will be achieved by using the software MAXQDA (version 24.9.1), as referenced by Kuckartz [27,28].

All results will be synthesized in a focus group in order to record and understand the care processes holistically and to classify them in the light of the existing evidence. The focus group will serve to validate and refine the results of the individual interviews, to contrast individual experiences within a group setting, and to enable in-depth exploration of shared experiences and interpretive patterns. Moreover, it supports the development of evidence-informed recommendations for care improvement. The synthesis will be published in scientific journals and made accessible to affected individuals and care providers to ensure broad dissemination and practical impact.

### Participants and Data Collection

The subprojects will be informed by a systematic literature review performed before the project (manuscript forthcoming).

*Subproject 1:* KVB claims data will be used for the analysis. The KVB data includes all claims for ambulatory physicians in the State of Bavaria made by insured persons residing in Bavaria. The analysis is based on the *Bavarian COVID-19 Cohort* which was established from anonymized billing records of all patients with statutory health insurance in Bavaria. Both general practitioners and medical specialists submit details on patient encounters, including treatments, diagnoses, and billing information, to the KVB in each quarter. The dataset includes pseudonymized patient identifiers, demographic variables (age, sex, region), diagnoses (International Classification of Diseases, 10th Revision, ICD-10 codes with diagnostic certainty) and billed services (EBM codes, specialty, and practice identifiers). This structure allows longitudinal tracking of health care utilization, comorbidities, and treatment patterns across cohorts.

Out of 9,202,930 individuals with any respiratory infection, 4,247,455 had a confirmed COVID-19 diagnosis and the remained any other respiratory infection. For the analysis, three cohorts were randomly drawn:

1. COVID-19 (2020–2022): 196, 000 individuals with a confirmed diagnosis of COVID-19, ie, *ICD* codes U07.1 or U08.9 or U09.9 (secured and suspected diagnoses)
2. Control (2020–2022): 140,000 individuals matched to the COVID-19 cohort by age and sex who received a confirmed ambulatory diagnosis of a respiratory infection (*ICD* J00*–J22*, J40*–J42*) in the same quarter.
3. Historical control (2019): 140,000 individuals with a confirmed respiratory infection (*ICD* J00*–J22*, J40*–J42*) diagnosed in 2019, matched by age and sex to the COVID-19 cohort

*Subproject 2:* Patients with PCC will be asked to participate in individual interviews about their experiences with health care delivery. Inclusion criteria comprise adult patients from the Munich region and the wider Bavaria area who either have a confirmed diagnosis of PCC or self-report having experienced PCC in the past or currently. Participants must have received or be receiving ambulatory treatment. Recruitment will be conducted through PCC support groups in Munich. These groups bring together individuals affected by PCC who meet regularly to exchange experiences and provide mutual support. A semistructured interview guideline will be developed and tested, taking into account individual experiences and care processes. Findings from the systematic review will be incorporated into the development of the guidelines. The guideline will establish a thematic framing and consists of all relevant topics that need to be addressed in the interview. It ensures better comparability of the data and structures the entire communication process [29]. Potential interview partners for the qualitative part of the project will be recruited utilizing a snowball sampling approach wherever possible [30]. This process follows the principle of maximum variation, aiming to capture a diverse range of perspectives and experiences. The qualitative interviews and the focus group will be conducted in person or via video conference. During the interview, patient journey maps will be developed [31,32]. The number of interviews is determined according to the methodological criterion of theoretical saturation [33]. The sample is expected to consist of around 20 to 25 participants. Should saturation be achieved earlier, indicated by the emergence of no new themes, a reduced number of interviews will be conducted. Conversely, if saturation is not attained, the sample size will be stepwise increased until the required number is reached.

The study will include a focus group, comprising 5‐10 participants. The discussion will take place face-to-face in a university meeting room and will be facilitated by two researchers. If an in-person participation is not possible due to severeness of disease, an additional online-based option will be made available. Results of both the data analysis and the synthesis of the individual interviews will be presented. Discussions will follow a semistructured guide with open-ended questions and are planned to last between 60 and 90 minutes. Participants will be selected from the pool of individuals who previously took part in the individual interviews, applying the same inclusion criteria. Recruitment will occur through renewed email contact. Group composition will be arranged to allow discussion of both shared and divergent care experiences. Participation will be voluntary, with withdrawal possible at any time without consequences. Before the discussion begins, sociodemographic information will be collected and written informed consent for participation and audio recording will be obtained on site. The moderator will lead the discussion, while the co-moderator will oversee time management, observe group dynamics, and take field notes. Data will be collected through audio recordings supplemented by field notes. All data will be pseudonymized, and deleted after transcription is completed.

### Data Analysis

#### Subproject 1

For the claims data, control groups will be matched by age, gender, and time of diagnosis (year/quarter). We will analyze the utilization patterns longitudinally for the cohorts and will focus specifically on the health care service billing group. We will estimate the incidence of PCC diagnoses (*ICD* codes: U09.9, U07.4), as well as newly occurring comorbidities, which may also be in the context of PCC. Expected outcomes are: (1) SARS-CoV-2 related symptoms, (2) newly developed diseases (cardiovascular diseases, autoimmune diseases, mental health diseases). Here, a special focus will be on diseases related to PCC, irrespective of a specific PCC diagnosis (eg, ICD-10 codes: R53, G93.3, F06.7, R43, M79.1, I26, R05, F48.0, F41) [4,34]. Health care utilization is analyzed using a *State Sequence Analysis* (SSA) [35]. Analyses are conducted for the cohort of SARS-CoV-2 infections and the control groups. In the analysis, the health care utilization for primary and specialist care as well as their respective diagnoses will be analyzed in the time before and after the SARS-CoV-2 infection. A specific focus will be on the appearance of a PCC diagnosis or diagnoses of PCC-related conditions. SSA identifies individual care trajectories represented as ordered sequences of quarterly states, where each state reflects contacts with general practitioners, COVID-19 specialists, or other specialists. Dissimilarities between trajectories are quantified using a theory-driven cost matrix, and clusters of patients with similar utilization patterns are derived through an unsupervised medoid-based clustering procedure. The optimal number of clusters is selected based on established quality indices (eg, average silhouette width). Factors associated with the resulting care pathway clusters are subsequently analyzed using multivariable regression models.

Additionally, the potential health care utilization depends on regional factors. Therefore, the place of residence of the cohort is recorded at the level of the districts and independent cities of Bavaria as well as the degree of urbanization of the municipality of residence. The regional distributions of the cohort and utilization patterns are visualized and analyzed using *geographical information systems* (GIS). Differences are analyzed using hierarchical regression models.

#### Subproject 2

All audio recordings from the interviews will be transcribed verbatim to ensure an accurate representation of participants’ perspectives. The evaluation process of the qualitative interviews employs thematic content analysis, as outlined by Mayring, to identify patterns and themes that emerge from the narratives [33]. This analytical process will involve both deductive category application based on the interview guide and inductive category development derived from the data itself. To support transparency, traceability, and efficient data management, MAXQDA (version 24.9.1) software will be used for coding and category refinement. Throughout the analysis, regular team discussions will be conducted to enhance reflexivity and ensure the reliability and coherence of the coding framework.

Furthermore, the results of the quantitative and qualitative subprojects are compared, and aspects of care are identified where there are divergences between the qualitative experience and the quantitative analyses. The prepared results of the care pathways of the claims data analysis and the experiences of the interview study are presented to the participants of the focus group and discussed. In the focus group, recommendations for action will be developed. The transcript will be analyzed using qualitative content analysis following Mayring’s approach [33], which enables a systematic, rule-guided development of categories and the stepwise interpretation of the material.

## Results

The study was funded by the German Federal Joint Committee Innovation Fund in October 2024 and is progressing according to the planned timeline, with data collection and analysis set to commence thereafter to generate meaningful results. Subproject 1: Statutory health insurance claims data covering the period from 2019 to 2022 were provided by the KVB. The first access to the routine data was in March 2025, with corrections made in April 2025. Analysis of the routine data began in July 2025 with quality control and descriptive analyses, with the first results expected by May 2026. Further analyses will be conducted until April 2027. Subproject 2: The recruitment of participants for the qualitative interviews and focus groups began in May 2025, and data collection and recruitment are expected to be finished by June 2026. As of manuscript submission, study preparation and ethics approvals have been completed, and 14 participants have been recruited for the qualitative interviews. First results of the qualitative interviews and focus group are expected in August 2026. The synthesis of the results and the development of recommendations are expected to be finished in August 2027.

## Discussion

This mixed-methods study will contribute to deepening and expanding the current state of research by combining systematic evidence synthesis, qualitative and quantitative approaches of subjective care experiences and objective care pathways of patients with PCC. The mixed-methods design was chosen to capture individual experiences of care that can be triangulated with the results of the routine data analysis, interpreted and embedded in the current state of research. The combination of different methods is a strength of this study, as it offers the opportunity to gain a deeper and more comprehensive understanding of ambulatory care practices for PCC. While there may be different descriptions or explanations, they complement each other and contribute to a broader picture [27,36].

It is expected that patients with PCC will show increased health care utilization. The wide range of symptoms observed in this population, combined with the heterogeneity of available health care services, presents a major challenge in identifying appropriate care pathways. Therefore, this study determines, in comparison with control cohorts, which services were utilized and what outcomes were achieved. In addition, it seeks to identify approaches that could improve care delivery. While the expected findings are currently limited to COVID-19 and PCC, they may offer valuable insights for managing other post-infectious conditions and for supporting patients with still persistent PCC symptoms. This could lead to a more efficient use of resources in the ambulatory sector and support earlier improvements in patients’ health. Through the integration of the systematic review, as well as subjective and objective care pathways and experiences, the study offers a substantial opportunity for an improved care provision.

As for all studies, some limitations need to be considered. First, in analyzing claims data, it must be acknowledged that clinical information is only available to a limited extent, as coding is primarily based on billing-related aspects. This can result in inaccuracies, particularly regarding *ICD* codes, due to the lack of mandatory coding standards, especially in general practice settings [37]. In addition, the dataset is limited to the region of Bavaria, which may introduce regional bias in observed health care utilization patterns. Temporal inaccuracies can also occur, as the recorded dates often reflect billing processes rather than the exact time of care provision [38].

Second, for the qualitative study, interviews capture subjective perspectives rather than objective realities. Recruitment via support groups and snowball sampling may lead to an overrepresentation of individuals who are more engaged or better connected within patient networks. Individuals with PCC who are less socially connected or socioeconomically disadvantaged may be underrepresented. While qualitative research does not aim for statistical representativeness, it seeks to provide in-depth insights into experiences, perceptions, and contextual factors [39]. The interview setting may introduce selectivity or artificiality, especially with personal topics like health. Moreover, the interpretative nature of the method involves a degree of subjectivity, and inherent trade-offs, such as between narrative depth and comparability which must be carefully managed [40]. The methodology for preparing and conducting the interviews will therefore be carefully designed to ensure consistency, taking into account all aspects of the research to enhance both the validity of the interviews and the overall understanding of the study. Furthermore, the focus only on patients in the Munich region may not fully capture health care experiences and developments in other geographical or more rural areas. A potential bias may also arise among focus group participants, as individuals involved in such networks may be more proactive or reflective about their situation.

Based on the expected results, this study is intended to provide concrete evidence for decision-makers in health care policy, particularly with regard to the future planning and organization of care structures. The more systematic inclusion of the patient-centered perspective can improve the relevance and effectiveness of future care concepts.

## Abbreviations

COREQ: Consolidated criteria for reporting qualitative research
GIS: Geographical information system
ICD-10: International Classification of Diseases, 10th Revision
KVB: Association of Statutory Health Insurance Physicians in Bavaria (Kassenärztliche Vereinigung Bayern)
PCC: Post-COVID-19 Condition
SARS-CoV-2: Severe Acute Respiratory Syndrome Coronavirus 2
SSA: State Sequence Analysis
STROBE: Strengthening the Reporting of Observational Studies in Epidemiology

## References

[1] (2024). WHO COVID-19 dashboard. World Health Organization.

[2] Chen C, Haupert SR, Zimmermann L, Shi X, Fritsche LG, Mukherjee B (2022). Global prevalence of post-coronavirus disease 2019 (COVID-19) condition or long covid: a meta-analysis and systematic review. J Infect Dis.

[3] O’Mahoney LL, Routen A, Gillies C (2023). The prevalence and long-term health effects of Long Covid among hospitalised and non-hospitalised populations: a systematic review and meta-analysis. EClinicalMedicine.

[4] (2025). Post COVID-19 condition (long COVID). World Health Organization.

[5] Lopez-Leon S, Wegman-Ostrosky T, Perelman C (2021). More than 50 long-term effects of COVID-19: a systematic review and meta-analysis. Sci Rep.

[6] Greenhalgh T, Sivan M, Perlowski A, Nikolich JŽ (2024). Long COVID: a clinical update. The Lancet.

[7] Natarajan A, Shetty A, Delanerolle G (2023). A systematic review and meta-analysis of long COVID symptoms. Syst Rev.

[8] Raveendran AV, Jayadevan R, Sashidharan S (2021). Long COVID: an overview. Diabetes Metab Syndr.

[9] Sudre CH, Murray B, Varsavsky T (2021). Attributes and predictors of long COVID. Nat Med.

[10] Luo D, Mei B, Wang P (2024). Prevalence and risk factors for persistent symptoms after COVID-19: a systematic review and meta-analysis. Clin Microbiol Infect.

[11] Su S, Zhao Y, Zeng N (2023). Epidemiology, clinical presentation, pathophysiology, and management of long COVID: an update. Mol Psychiatry.

[12] Castriotta L, Onder G, Rosolen V (2024). Examining potential Long COVID effects through utilization of healthcare resources: a retrospective, population-based, matched cohort study comparing individuals with and without prior SARS-CoV-2 infection. Eur J Public Health.

[13] DeVoss R, Carlton EJ, Jolley SE, Perraillon MC (2025). Healthcare utilization patterns before and after a long COVID diagnosis: a case-control study. BMC Public Health.

[14] Tene L, Bergroth T, Eisenberg A, David SSB, Chodick G (2023). Risk factors, health outcomes, healthcare services utilization, and direct medical costs of patients with long COVID. Int J Infect Dis.

[15] Huang BZ, Creekmur B, Yoo MS, Broder B, Subject C, Sharp AL (2022). Healthcare utilization among patients diagnosed with COVID-19 in a large integrated health system. J Gen Intern Med.

[16] Koumpias AM, Schwartzman D, Fleming O (2022). Long-haul COVID: healthcare utilization and medical expenditures 6 months post-diagnosis. BMC Health Serv Res.

[17] Musheyev B, Boparai MS, Kimura R (2023). Longitudinal medical subspecialty follow-up of critically and non-critically ill hospitalized COVID-19 survivors up to 24 months after discharge. Intern Emerg Med.

[18] Tartof SY, Malden DE, Liu ILA (2022). Health care utilization in the 6 months following SARS-CoV-2 infection. JAMA Netw Open.

[19] Gamillscheg P, Łaszewska A, Kirchner S, Hoffmann K, Simon J, Mayer S (2024). Barriers and facilitators of healthcare access for long COVID-19 patients in a universal healthcare system: qualitative evidence from Austria. Int J Equity Health.

[20] Gardner E, Lockrey A, Stoesser KL (2024). Challenges in receiving care for long COVID: a qualitative interview study among primary care patients about expectations and experiences. Ann Fam Med.

[21] Schmachtenberg T, Königs G, Dragaqina A (2023). “There is no one who helps you with it”: experiences of people with long COVID regarding medical care, therapeutic measures, and barriers in the German healthcare system: results of a qualitative study with four focus groups. BMC Health Serv Res.

[22] MacEwan SR, Rahurkar S, Tarver WL (2024). Patient experiences navigating care coordination for long COVID: a qualitative study. J GEN INTERN MED.

[23] Turk F, Sweetman J, Chew-Graham CA (2024). Accessing care for long Covid from the perspectives of patients and healthcare practitioners: a qualitative study. Health Expect.

[24] Tong A, Sainsbury P, Craig J (2007). Consolidated criteria for reporting qualitative research (COREQ): a 32-item checklist for interviews and focus groups. Int J Qual Health Care.

[25] von Elm E, Altman DG, Egger M (2014). The Strengthening the Reporting of Observational Studies in Epidemiology (STROBE) Statement: guidelines for reporting observational studies. Int J Surg.

[26] Page MJ, McKenzie JE, Bossuyt PM (2021). The PRISMA 2020 statement: an updated guideline for reporting systematic reviews. BMJ.

[27] Fetters MD, Curry LA, Creswell JW (2013). Achieving integration in mixed methods designs-principles and practices. Health Serv Res.

[28] Kuckartz U (2014). Mixed Methods: Methodologie, Forschungsdesigns Und Analyseverfahren.

[29] Misoch S (2019). Qualitative Interviews: Walter de Gruyter GmbH & Co KG.

[30] Heckathorn DD (1997). Respondent-driven sampling: a new approach to the study of hidden populations. Soc Probl.

[31] Trebble TM, Hansi N, Hydes T, Smith MA, Baker M (2010). Process mapping the patient journey: an introduction. BMJ.

[32] Davies EL, Bulto LN, Walsh A (2023). Reporting and conducting patient journey mapping research in healthcare: a scoping review. J Adv Nurs.

[33] Mayring P, Flick U, Keupp H, S W (1991). Handbuch qualitative Forschung : Grundlagen, Konzepte, Methoden und Anwendungen.

[34] Donnachie E, Hapfelmeier A, Linde K (2022). Incidence of post-COVID syndrome and associated symptoms in outpatient care in Bavaria, Germany: a retrospective cohort study using routinely collected claims data. BMJ Open.

[35] Gabadinho A, Ritschard G, Mueller NS, Studer M (2011). Analyzing and visualizing state sequences in R with TraMineR. J Stat Softw.

[36] Tariq S, Woodman J (2013). Using mixed methods in health research. JRSM Short Rep.

[37] Slagman A, Hoffmann F, Horenkamp-Sonntag D, Swart E, Vogt V, Herrmann WJ (2023). Analyse von Routinedaten in der Gesundheitsforschung: Validität, Generalisierbarkeit und Herausforderungen. Z Allg Med.

[38] Swart E, Gothe H, Geyer S (2015). Good Practice of Secondary Data Analysis (GPS): guidelines and recommendations. Gesundheitswesen.

[39] Pyo J, Lee W, Choi EY, Jang SG, Ock M (2023). Qualitative research in healthcare: necessity and characteristics. J Prev Med Public Health.

[40] Hohl J (2000). Das qualitative interview. J Public Health (Oxf).

